# ATP-sensitive potassium channels modulate *in vitro* tocolytic effects of β_2_-AR agonists on uterine muscle rings in rats in early but not in late pregnancy

**DOI:** 10.3325/cmj.2015.56.114

**Published:** 2015-04

**Authors:** Norbert Lovasz, Andrea Koncz, Dora Domokos, Robert Gaspar, György Falkay

**Affiliations:** Department of Pharmacodynamics and Biopharmacy, Faculty of Pharmacy, University of Szeged, Szeged, Hungary

## Abstract

**Aim:**

To investigate whether ATP-sensitive potassium (K_ATP_) channels modulate the tocolytic effect of β_2_-AR agonists (ritodrine and salmeterol) in early-pregnant (day 6) and late-pregnant (day 22) rat uterus *in vitro*, in order to examine the relation between the K_ATP_ channel sulphonylurea-binding regulatory subunit (SUR) expression and pharmacological reactivity of β_2_-AR agonists.

**Methods:**

The tocolytic effects of ritodrine and salmeterol (10^-10^-10^-5^ M) on spontaneous rhythmic contractions were investigated cumulatively, alone, or in the presence of the K_ATP_ channel blocker glibenclamide (10^-6^ M) and the K_ATP_ channel opener pinacidil (10^-9^-10^-7^ M) after 5-min preincubation.

**Results:**

β_2_-AR agonist induced myometrial relaxation was inhibited by glibenclamide and enhanced by pinacidil on day 6, when SUR1 expression levels were high. Neither glibenclamide nor pinacidil mediated tocolytic effect was measured on day 22.

**Conclusion:**

Low expression of the K_ATP_ channels at the end of gestation may facilitate enhanced excitability and contractility in the rat myometrium. The combination of a betamimetic and a K_ATP_ channel opener will therefore not be of therapeutic relevance in the treatment of preterm delivery.

A number of agents have tocolytic effect, including β_2_-adrenergic receptor (β_2_-AR) agonists, magnesium sulfate, prostaglandin synthesis inhibitors, Ca^2+^-channel blockers, nitrogen monoxide donors, and oxytocin receptor antagonists (1). β_2_-AR agonists (such as salmeterol, terbutaline, fenoterol, hexoprenaline, and ritodrine) delay preterm labor for at least 48 hours, which is why they are the drugs of choice in the treatment of preterm labor (2). Of all tocolytics in use, β-mimetics have the most undesirable side-effect profile. The most serious reported side-effects associated with the administration of β_2_-AR agonists are pulmonary edema, hypotension, and tachycardia (3,4). Promising new therapeutic approach for the treatment of preterm delivery is the combination β_2_-AR agonists and 17alpha-hydroxyprogesterone (5) or Ca^2+^-channel blocker nifedipine (6).

Adenosine triphosphate (ATP)-sensitive potassium channels (K_ATP_ channels) are involved in β-AR agonists-induced smooth muscle relaxation in pulmonary vasorelaxation in the rat (7), vasodilatation in the rat diaphragmatic microcirculation (8), vasorelaxation in the rat mesenteric artery (9), detrusor muscle relaxation in the rat (10), and myometrial relaxation in non-pregnant buffaloes (11). K_ATP_ channels are formed by a combination of two types of subunits, the pore-forming inwardly rectifying subunit (Kir_6x_) and the sulphonylurea-binding regulatory subunit (SUR) (12). We earlier reported (13) that SUR subunits, SUR1 and SUR2, are both expressed in the rat uterus during gestation. SUR1 expression was elevated in the early pregnancy (day 6) and then dramatically decreased from day 8 to term, while the level of SUR2 subunit remained unchanged.

The aim of the present study was to investigate the role of the K_ATP_ channel in β_2_-AR agonist-induced myometrial relaxation. We studied the tocolytic effects of β_2_-AR agonists (salmeterol, ritodrine) in the presence of glibenclamide (K_ATP_ channel blocker) and pinacidil (K_ATP_ channel opener) in early pregnant (day 6) and late pregnant (day 22) rats *in vitro*, in order to clarify the relation between SUR1 expression and pharmacological reactivity of β_2_-AR agonists.

## Materials and methods

### Mating of the animals

The study was approved by the Hungarian Ethics Committee for Animal Research (registration number: IV/01758-2/2008) and the animals were treated in accordance with the European Communities Council Directives (86/609/ECC) and the Hungarian Act for the Protection of Animals in Research (XXVIII.tv.32.§). Sprague-Dawley rats (Charles-River Laboratories, Budapest, Hungary) were kept at 22 ± 3°C with relative humidity of 30%-70% and 12/12 h light/dark cycle. They were fed a standard rodent pellet diet (Charles-River Laboratories) with tap water available *ad libitum*.

Mature female (180-200 g) and male (240-260 g) rats were mated in a special mating cage with a metal door separating the rats of different sex, which is open when the rats are allowed to mate. Four to five hours after the potential mating, vaginal smears were taken from the female rats. The female rats in which sperm cells were microscopically detected (magnification of 1200 times) and those in whom smears could not have been taken because of a vaginal sperm plug were regarded as first-day pregnant animals (13).

### Uterus preparation

The rats were euthanized by CO_2_ inhalation and the uteri were removed on the 6th and 22nd day of pregnancy. Muscle rings 5 mm long were sliced from the uterine horns and mounted vertically in an organ bath containing 10 mL of de Jongh solution (composition: 137 mM NaCl, 3 mM KCl, 1 mM CaCl_2_, 1 mM MgCl_2_, 12 mM NaHCO_3_, 4 mM NaH_2_PO_4_, 6 mM glucose, pH = 7.4). The organ bath was maintained at 37°C, and carbogen (95% O_2_ + 5% CO_2_) was bubbled through it. After mounting, the rings were equilibrated for about 1 h before the experiments, with a solution change every 15 min. The initial tension of the preparation was set to about 1.25 g, which was relaxed to about 0.5 g at the end of equilibration. The tension of the myometrial rings was measured with a gauge transducer (SG-02; Experimetria Ltd, Budapest, Hungary) and recorded with a SPEL Advanced ISOSYS Data Acquisition System (Experimetria Ltd) (13).

### *In vitro* studies

The tissue samples were incubated for 5 min and the tocolytic effect of β_2_-AR agonists ritodrine and salmeterol (10^−10^-10^−5^ M) on spontaneous rhythmic contractions was investigated cumulatively, alone, or in the presence of K_ATP_ channel blocker glibenclamide (10^−6^ M) or K_ATP_ channel opener pinacidil (10^−9^-10^−7^ M). Following the addition of each dose of β_2_-AR agonist, the changes were recorded for 300 s. Concentration-response curves were fitted and areas under curves (AUCs) were determined. Statistical analysis was carried out with the Prism 5.0 (Graphpad Software Inc., San Diego, CA, USA). From the AUC values, maximum inhibitory effects (E_max_) of β_2_-AR agonists on a given day of pregnancy were calculated and the concentrations eliciting 50% of the maximum inhibition of uterine contraction (EC_50_) were calculated. Data were analyzed with the ANOVA Neuman-Keuls test. The alpha value was 0.05. The variances were constant and the distribution was normal.

## Results

### Both glibenclamide and pinacidil influenced the effect of ritodrine and salmeterol

**Figure 1:**
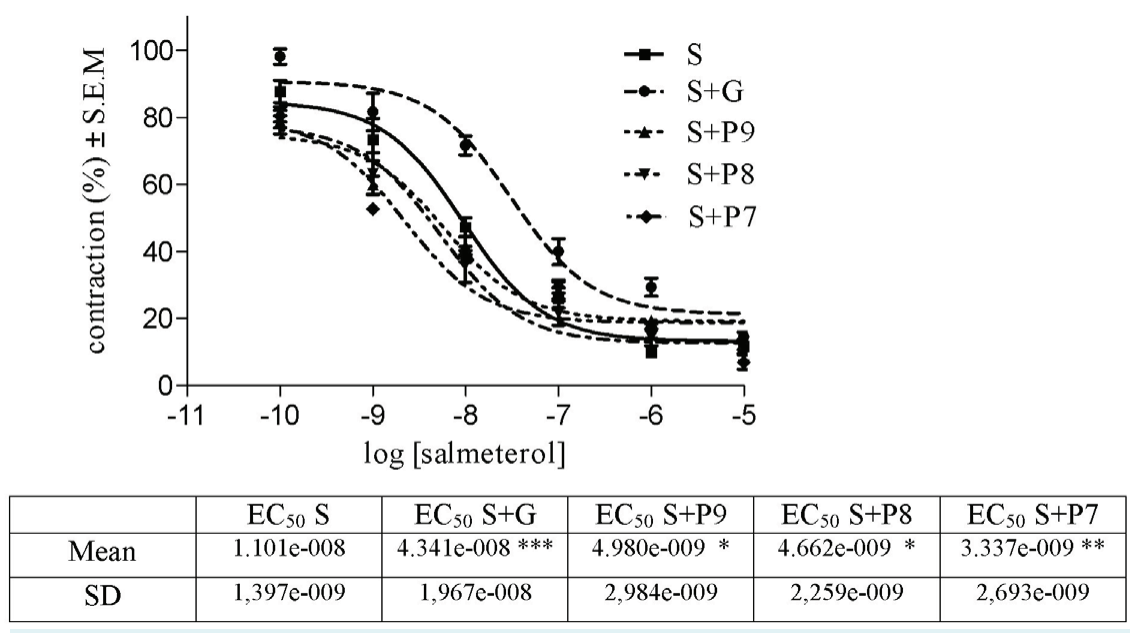
The tocolytic effect of β_2_-AR agonist salmeterol alone (10^−10^-10^−5^ M) (**S**) and in the presence of glibenclamide (S+G) and pinacidil (10^−9^ M: S+P9, 10^−8^ M: S+P8 and 10^−7^ M: S+P7) in the myometrium of 6-day-pregnant rat *in vitro* (mean± SE). The table shows EC_50_ data (mean ± SD, n = 6). Abbreviations: P9: pinacidil 10^−9^M, P8: pinacidil 10^−8^M, P7: pinacidil 10^−7^M, EC_50:_ the concentrations eliciting 50% of the maximum inhibitions of uterine contraction, SE: standard error, SD: standard deviation.

**Figure 2:**
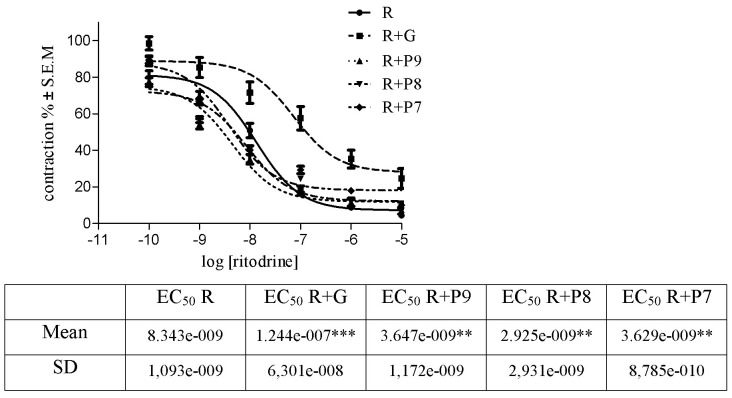
The tocolytic effect of β_2_-AR agonist ritodrine alone (10^−10^-10^−5^ M) (**R**) and in the presence of glibenclamide (R+G) and pinacidil (10^−9^ M: R+P9, 10^−8^ M: R+P8 and 10^−7^ M: R+P7) in the myometrium of 6-day-pregnant rat *in vitro* (mean± SE). The table shows EC_50_ data (mean ± SD, n = 6). See Figure 1 for abbreviations.

Glibenclamide blocked the tocolytic effects of β_2_-AR agonists; the dose-response curves shifted to the right, and the EC_50_ values of β_2_-AR agonists significantly increased. Pinacidil enhanced the tocolytic effects of β_2_-AR agonists; the dose-response curves shifted to the left and the EC_50_ values of β_2_-AR agonists significantly decreased (Figure 1 and 2).

### Neither glibenclamide nor pinacidil influenced the effects of the β_2_-AR agonists on the 22 day pregnant uterus

**Figure 3:**
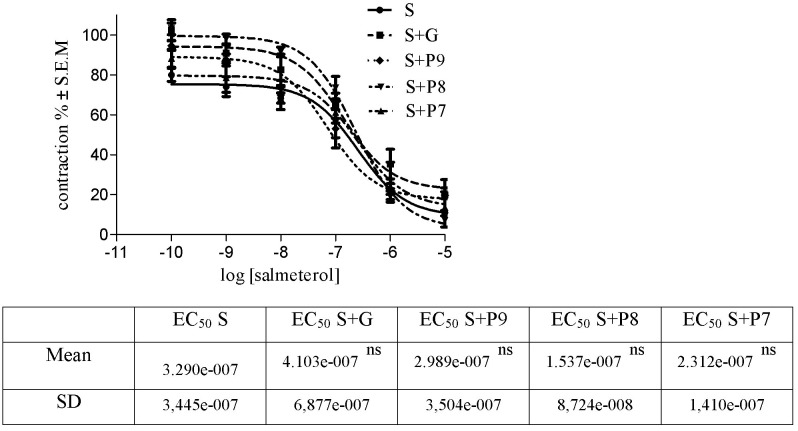
The tocolytic effect of β_2_-AR agonist salmeterol alone (10^−10^-10^−5^ M) (**S**), in the presence of glibenclamide (S+G), and pinacidil (10^−9^ M: S+P9, 10^−8^ M: S+P8 and 10^−7^ M: S+P7) in the myometrium of 22-day-pregnant rat *in vitro* (mean± SE). The table shows EC_50_ data (mean ±SD, n = 6). See Figure 1 for abbreviations.

**Figure 4:**
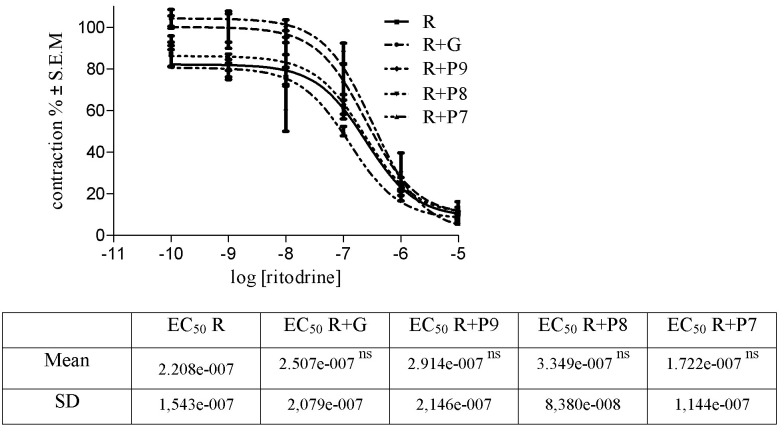
The tocolytic effect of β_2_-AR agonist ritodrine (10^−10^-10^−5^ M) alone (**R**) in the presence of glibenclamide (R+G) and pinacidil (10^−9^ M: R+P9, 10^−8^ M: R+P8 and 10^−7^ M: R+P7) in the myometrium of 22-day-pregnant rat *in vitro* (mean± SE). The table shows EC_50_ data (mean ± SD, n = 6). See Figure 1 for abbreviations.

The uterus-relaxant effects of ritodrine and salmeterol (10^−10^-10^−5^ M) on the 22-day-pregnant rat uterus were investigated in the presence of glibenclamide (10^−6^ M) or different doses of pinacidil (10^−9^, 10^−8^ and 10^−7^ M) (Figure 3 and 4).

## Discussion

Preterm delivery is one of the greatest challenges in obstetrical practice. The factors regulating myometrial function during pregnancy and labor are poorly understood. Understanding of these processes at cellular and molecular levels is essential for development of new therapeutic strategies. β_2_-ARs affect the contractility of the pregnant uterus which is why they are used for the treatment of premature labor.

K_ATP_ channels are large hetero-octameric complexes containing four subunits from the inwardly rectifying K^+^ channel family (Kir_6.x_: either Kir_6.1_ or Kir_6.2_) and four SUR subunits from the ABC transporter family: ABCC8 (SUR1) and ABCC9 (SUR2). SUR2 has two different isoforms, SUR2A and SUR2B, which are splicing variants. Both types of subunits, SURs and Kir_6.x_ are necessary for the channel function. Kir_6.x_ comprises the channel component of the K_ATP_, while the SURs are responsible for the ATP sensitivity, pharmacological properties, and trafficking of this channel (14-18). K_ATP_ channels have different molecular structure, due to the heterologous expression of the Kir_6.x_ and SUR subunits. This leads to different combinations and creates different types of K_ATP_ channels with distinct electrophysiological properties and pharmacological sensitivities.

We found earlier (13) that both SUR1 and SUR2 subunits were expressed in the rat uterus during gestation: SUR1 was markedly increased on day 6 and dramatically decreased from day 8 to term, while the level SUR2 subunit remained low during the entire gestation. The present study showed that K_ATP_ channels modulated the tocolytic effect of β_2_-AR agonists in the rat on day 6 of gestation. We clearly demonstrated that in the early gestation, when SUR1 level was elevated, tocolytic effect of β_2_-AR agonist was inhibited by glibenclamide and potentiated by pinacidil, while at the end of gestation, when SUR1 level was decreased, it was influenced by neither glibenclamide nor pinacidil. It can be concluded that the mediation effect of the K_ATP_ channels on the efficacy of the β_2_-AR agonist depends on the expression of the SUR1 subunit of the K_ATP_ channels. We had earlier demonstrated that the tocolytic effects of the β_2_-AR agonists in the rat significantly decreased in late (days 15, 18, 20, and 22 of gestation) compared to early gestation (19). This phenomenon could be explained by a decrease in the β_2_-AR function, which is partially controlled by β-adrenergic kinase, the estrogen/progesterone levels, and G-protein-coupled receptor kinases (20-22). Our results indicate that there are other mechanisms that decrease the tocolytic effect of β_2_-AR agonists at the end of gestation. The low levels of K_ATP_ channels at the end of gestation may facilitate the enhanced excitability and contractility of the myometrium, which is one of the reasons for the decreased efficacy of the betamimetics. It was shown (23) that SUR1 expression in the human myometrium was decreased in late pregnancy compared with non-pregnant women. Moreover, low levels of Kir_6.1_ and Kir_6.2_ subunits were determined at the end of gestation. Since open K_ATP_ channels draw the cell membrane potential closer to the K^+^ equilibrium potential, K_ATP_ channels are closely involved in reducing cellular excitability and contractility. The combination of betamimetics with a K_ATP_ channel opener will therefore not have any therapeutic relevance in the treatment of preterm birth. However, we can hypothesize that this combination may be used as a uterus relaxant in the early gestation (eg, habitual abortion).
